# 2D/0D graphene hybrids for visible-blind flexible UV photodetectors

**DOI:** 10.1038/s41598-017-05981-y

**Published:** 2017-07-17

**Authors:** Hiroyuki Tetsuka

**Affiliations:** Frontier Research-Domain, Toyota Central R&D Labs., Inc., 41-1 Yokomichi, Nagakute, Aichi 480-1192 Japan

## Abstract

Nitrogen-functionalized graphene quantum dots (NGQDs) are attractive building blocks for optoelectronic devices because of their exceptional tunable optical absorption and fluorescence properties. Here, we developed a high-performance flexible NGQD/graphene field-effect transistor (NGQD@GFET) hybrid ultraviolet (UV) photodetector, using dimethylamine-functionalized GQDs (NMe_2_-GQDs) with a large bandgap of ca. 3.3 eV. The NMe_2_-GQD@GFET photodetector exhibits high photoresponsivity and detectivity of ca. 1.5 × 10^4^ A W^–1^ and ca. 5.5 × 10^11^ Jones, respectively, in the deep-UV region as short as 255 nm without application of a backgate voltage. The feasibility of these flexible UV photodetectors for practical application in flame alarms is also demonstrated.

## Introduction

Graphene has important potential for use in highly efficient photodetectors because of its combination of broadband optical absorption and high carrier mobility^1^. However, the use of pristine graphene in photodetectors, for which spectral colour selectivity is desired, is hindered by its gapless nature, and by its weak and flat light absorption over a broad wavelength range. To overcome this shortcoming, the fabrication of hybrid photodetectors that can combine the advantages of graphene and a semiconductor material with a tunable bandgap presents the most promising approach. Hybrid graphene photodetectors with various photo-absorbing media have been studied for detection of ultraviolet (UV), visible, and near-infrared (NIR) light. An NIR photodetector with a photoresponsivity as high as ca. 10^6^ A W^−1^ has been fabricated by hybridizing graphene with PbS nanocrystals^2^. Integration of plasmonic Au nanorods has also provided NIR light detection with photoresponsivity of 4 × 10^4^ A W^−1^ at 1,310 nm via the hot carrier injection effect^3^. Visible light detection with photoresponsivity of 180 A W^−1^ at 400–800 nm was achieved by the combination of graphene with organometallic halide perovskites^4^. More recently, a hybrid photodetector that exhibits a full-colour optical bandwidth was produced through immobilization of rhodamine-based organic dye molecules with red, green, or blue light absorption profiles onto graphene, whereby photoresponsivity of 10^3^ A W^−1^ was obtained^5^. Such photodetectors’ performance for visible and NIR light detection compares favourably with that of the conventional compound semiconductor-based photodetectors^6–9^. In contrast to visible and NIR light detection, no efficient visible-blind UV detection photodetector has yet been developed. For instance, hybrid structures that use wide-bandgap semiconductors (ZnO nanocrystals or TiO_2_ nanowires) enable UV light detection^10, 11^. However, the ZnO/graphene hybrid photodetectors were not visible-blind. The TiO_2_/graphene hybrid photodetectors possessed low photoresponsivity of ca. 0.5 A W^−1^. Their performance is inferior to those of pristine wide-bandgap semiconductor-based visible-blind UV photodetectors^12, 13^.

We have also recently produced a high-performance hybrid photodetector with photoresponsivity of 4 × 10^4^ A W^−1^ in the deep-UV to NIR wavelength region that uses a 2D/0D graphene hybrid consisting of graphene and nitrogen-functionalized graphene quantum dots (NGQDs) with tunable bandgaps^14, 15^. The high photoresponsivity was a consequence of strong light absorption and long-life photogenerated carriers in the NGQDs^16, 17^. In addition to strong light absorption, NGQDs have bandgap tunability that covers a range of absorption from deep-UV to IR. They can thereby be extended to exploit visible-blind UV photodetection. This report describes the performance of a graphene/NGQD hybrid photodetector for visible-blind flexible UV photodetectors. Dimethylamine-functionalized graphene quantum dots (NMe_2_-GQDs) with a large bandgap of ca. 3.3 eV were used for the NGQD layer to enable visible-blind UV light detection. The photosensitivity and detectivity of the NMe_2_-GQD@GFET (graphene field-effect transistor) was investigated. The feasibility of these flexible photodetectors for practical application for flame detection was demonstrated.

## Results and Discussion

**Figure 1:**
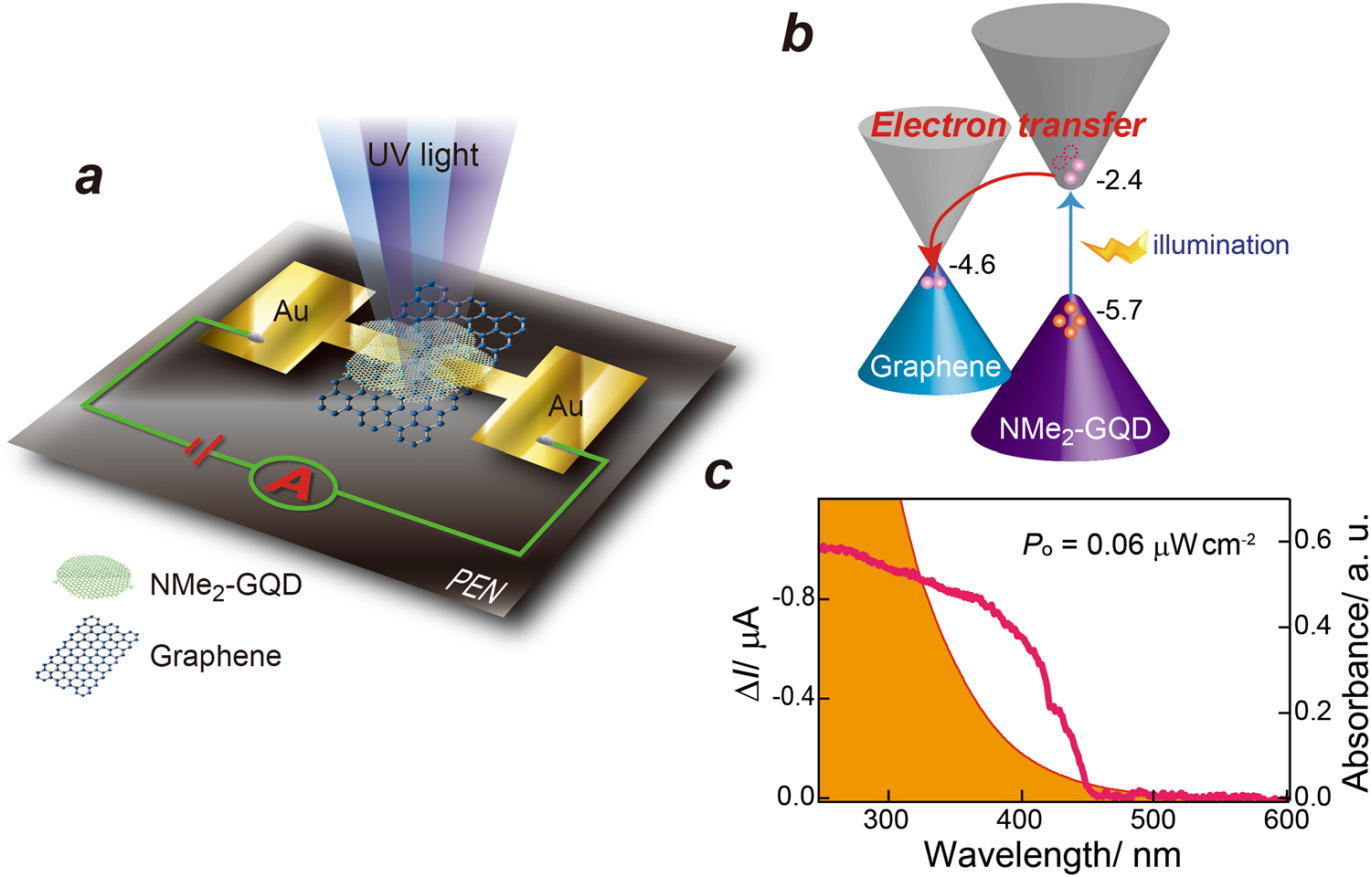
(**a**) Schematic representation of the NMe_2_-GQD@GFET hybrid photodetector. (**b**) Schematic diagram of the energy level alignment for the photodoping effect under illumination. Photoexcited electrons in the NMe_2_-GQDs are transferred to the graphene layer. (**c**) Spectral response (left axis) from the NMe_2_-GQD@GFET photodetector (*V*_SD_ = 0.1 V, *V*_G_ = 0 V, 0.06 μW cm^−2^). The solid orange spectrum (right axis) corresponds to the UV-vis absorption spectrum of a NMe_2_-GQD solution.

Figure 1a portrays a schematic diagram of the top-contact graphene/NGQD hybrid UV photodetector. The carrier transport channel was graphene on top of the 188-μm-thick polyethylene naphthalate (PEN) substrate. The photon-absorbing layer comprised NMe_2_-GQDs with an energy gap of 3.3 eV, which enables visible-blind UV light detection. HRTEM observations indicated that the NMe_2_-GQDs had diameters of ca. 5 nm (Fig. S1a). AFM observations revealed most of the NMe_2_-GQDs as ca. 1.0 nm thick, which corresponds to 1–2 layers of functionalized GQDs (Fig. S1b and c). The high-resolution N 1 s XPS spectrum of the NMe_2_-GQDs revealed that nitrogen atoms were predominantly engaged in C–N bonding (400.1 eV), which is characteristic of primary dimethyl amines bonded to graphene (Fig. S1d). The band alignment at the graphene/NMe_2_-GQD interface under light irradiation is illustrated schematically in Fig. 1b. The lowest unoccupied molecular orbital (LUMO) level of NMe_2_-GQDs was estimated by subtracting the band gap (Fig. S1e) from the highest occupied molecular orbital (HOMO) level. The HOMO level was measured using ultraviolet photoelectron yield spectroscopy (Fig. S1f). Incident photons excite the ground-state electrons of the NMe_2_-GQDs into excited states. Electron-hole pairs are then generated. The photoexcited carriers are separated at the NMe_2_-GQD/graphene interface because of the internal built-in electric field. Only the electrons are swept into the graphene layer driving efficient charge separation and transfer, whereas the photoexcited holes remain in the NMe_2_-GQD layer.

The spectral response was measured. It corresponded well with typical absorbance behaviour for NMe_2_-GQDs, i.e., a steep rising edge positioned at ca. 420 nm. Figure 1c shows that the photodetector is exclusively sensitive to UV light. It is almost blind to visible light, although the NMe_2_-GQDs have an absorption tail in the visible light range (>450 nm), presumably because the localized edge states or surface states in the NMe_2_-GQDs. The edge states or surface states that contribute to light absorption of NMe_2_-GQDs in the longer wavelength range have little contribution to the photocurrent because the carriers on these states are highly localised. Moreover, they recombine shortly after generation. In contrast, the shorter wavelength light offers sufficient energy to excite the carriers to the LUMO energy level of the NMe_2_-GQDs, generating an efficient photocurrent^18^. A response cut-off wavelength was ca. 430 nm. The UV-to-visible rejection ratio (Δ*I*370/Δ*I*450 nm, where Δ*I* (=*I*_light_ − *I*_dark_) is the photocurrent)^12, 13^ of the photodetector is over two orders of magnitude (>5 × 10^2^), as shown in the response spectrum, which indicates that the photodetector exhibits a high signal-to-noise ratio. In fact, a negative value of Δ*I* was detected, as discussed below.

**Figure 2:**
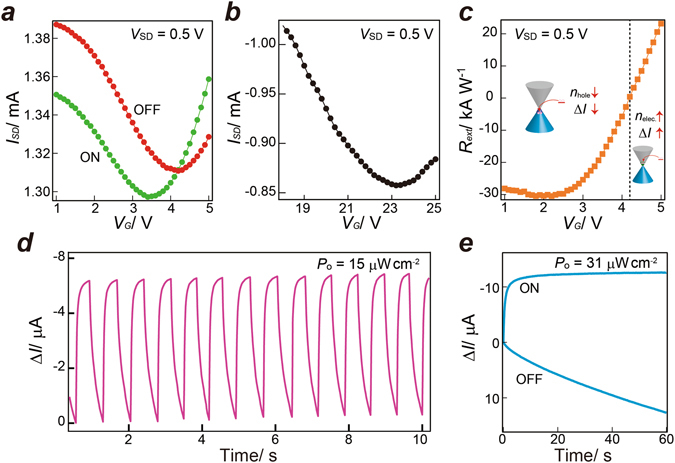
(**a**) *I*_SD_–*V* curve (*V*_SD_ = 0.5 V) for the ON (405 nm, 18 μW cm^−2^) and OFF states of a NMe_2_-GQD@GFET photodetector. (**b**) *I*_SD_–*V* curve (*V*_SD_ = 0.5 V) for a pristine GFET. (**c**) Photoresponsivity as a function of gate voltage. Insets show the direction of electron transfer from the NMe_2_-GQDs to graphene for the hole and electron conduction regimes in graphene under illumination (405 nm, 18 μW cm^−2^). (**d**) Temporal photocurrent response of the NMe_2_-GQD@GFET photodetector (*V*_SD_ = 0.1 V, *V*_G_ = 0 V, 405 nm, 15 μW cm^−2^). (**e**) Transient response dynamics for the NMe_2_-GQD@GFET photodetector (*V*_SD_ = 0.1 V, *V*_G_ = 0 V, 405 nm, 31 μW cm^−2^).

We further characterized the NMe_2_-GQD@GFET hybrid photodetector by measuring the transfer curve upon illumination. Figure 2a and b present the transfer characteristics (*V*_SD_ = 0.5 V) for the NMe_2_-GQD@GFET hybrid photodetector and a pristine GFET. The Dirac point (charge neutrality point, *V*_D_) from the pristine GFET was observed at ca. 23.5 V, implying unintentional hole doping from contamination, lithography processes, and/or surface oxygen-related adsorbates^19^. After hybridization with the NMe_2_-GQDs, *V*_D_ shifted to ca. 4.2 V, which suggests that electrons were transferred from the NMe_2_-GQDs to graphene. This transferral is consistent with the higher work function of NMe_2_-GQDs. Upon irradiation (405 nm, 18 μW cm^−2^) of the NMe_2_-GQD@GFET hybrid photodetector, *V*_D_ shifts to a lower voltage, which suggests that photoexcited electrons are transferred to the graphene. From the transfer curves measured in the absence of light, it is estimated that the field-effect mobility for holes is ca. 985 and 1,575 cm^2^ V^−1^ s^−1^, respectively, for the GFET and the NMe_2_-GQD@GFET. The hole-carrier mobility is increased by the incorporation of NMe_2_-GQDs, which is contrary to previously observed trends for graphene hybrid phototransistors, which exhibit decreased carrier mobility because of disorder or defects induced by hybridization^2, 10^. The increase in carrier mobility might be attributable to an increase in the density of states near the Fermi level induced by the widely distributed π orbitals of the NMe_2_-GQDs.

When the NMe_2_-GQD@GFET is operated at *V*_G_ = 0 V, the major carriers in the graphene layer are holes. The holes are compensated by the transferred electrons, which engenders decreased conductance and a negative *ΔI* (Fig. 1c). The back-gate dependence of the photoresponsivity (Fig. 2c) is also consistent with this trend. A clear carrier type and concentration dependence of the external photoresponsivity *R*_ext_ (*R*_ext_ = *ΔI*/*P*_light_, where *P*_light_ is the incident optical power density) were observed. *R*_ext_ (*R*_ext_ = *ΔI*/(*P*_light_ $\bullet$ *A*_PD_/*A*_light_), where *A*_light_ is the light spot area) was calculated with a scaling factor (*A*_PD_/*A*_light_) that takes into account that only a fraction of the optical power density impinges on the photodetector. In the hole-conduction region (*V*_G_ < 4.2 V), the holes are compensated by the photoinduced electron carriers, which engenders decreased conductance and a negative *R*_ext_. In contrast, the photoinduced electron carriers raise the electron concentration, which engenders increased conductance and a positive *R*_ext_ in the electron-conduction region (*V*_G_ > 4.2 V).

We investigated the dynamic performance of the NMe_2_-GQD@GFET hybrid photodetector by measuring its response time. Figure 2d shows the on/off photocurrent of the photodetector at an incident power density of 15 μW cm^−2^ (*V*_G_ = 0 V, *V*_SD_ = 0.1 V, 405 nm. The temporal photoresponse was measured at the on/off cycle of 1 s. The photocurrent level is well retained, demonstrating good reliability and reversibility of NMe_2_-GQD@GFET hybrid photodetector. Figure 2e gives the transient response dynamics. The rise time was ca. 3.7 s (which corresponds to a rise of ca. 90%). The decay trace can be fitted by a double exponential; *ΔI*_DS_ = *ΔI*_1_[1 − exp(−*t*/*τ*_1_)] + *ΔI*_2_[1 − exp(−*t*/*τ*_2_)]^10, 20^. Time constants *τ*_1_ and *τ*_2_ are, respectively, 22 s and 77 s. This slow decay is probably associated with the multiplicity of carrier traps in NMe_2_-GQDs from quantum confinement and different localized states in the π-π* gap^21^. The short relaxation time *τ*_1_ corresponds to the lifetime of the holes trapped in the NMe_2_-GQDs, whereas the long relaxation time *τ*_2_ represents the time duration for the charge transfer and transportation in NMe_2_-GQDs layer. The carrier transit time *τ*_transit_ (*τ*_transit_ = *L* ^2^⁄*μ*∙*V*_DS_) is estimated as 1.3 ns (based on a carrier mobility (*μ*) of 1,575 cm^2^ V^−1^ s^−1^, a channel length (*L*) of 10 μm, and a bias (*V*_DS_) of 0.5 V), thereby the photoconductive gain *G* (*G* = τ_life_/τ_transit_)^22^ is between 1.7 × 10^10^ and 5.9 × 10^10^, respectively using lifetimes of 22 s and 77 s. The high photoconductive gain is attributed to the cooperative effect of long hole-carrier lifetime in the NMe_2_-GQDs and the high carrier mobility of graphene. Multiple carrier circulation occurs in the graphene channel, depending on the ratio of the photoexcited holes relaxation time in the NMe_2_-GQDs layer to the carrier transit time in the graphene layer. Because the photogenerated holes remain trapped in the NMe_2_-GQDs, the positively charged NMe_2_-GQDs induce negative carriers in the graphene sheet through capacitive coupling. As long as the NMe_2_-GQDs remain positively charged, negative charges in the graphene sheet are highly recirculated because of their high carrier mobility, resulting in high photoconductive gain^2^.

**Figure 3:**
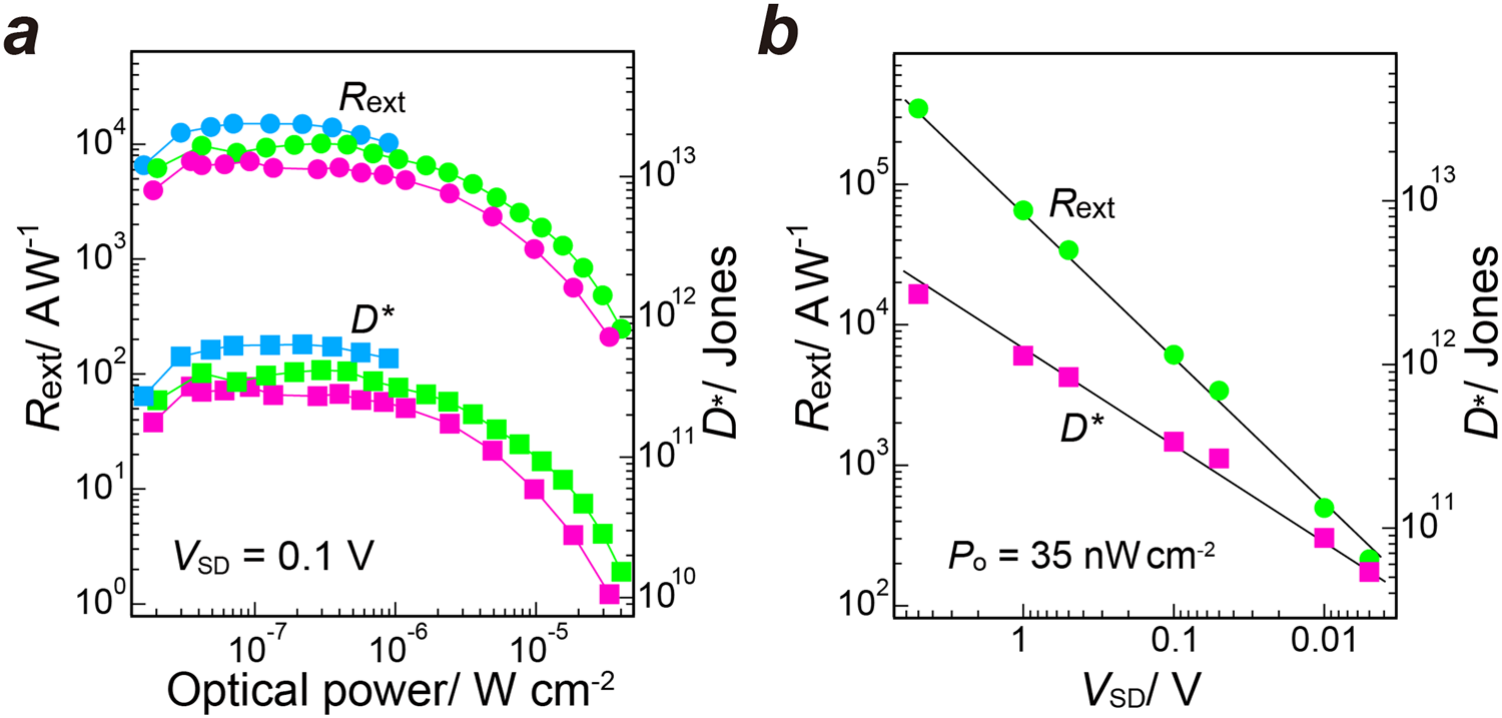
(**a**) Photoresponsivity and specific detectivity of the NMe_2_-GQD@GFET hybrid photodetector (*V*_SD_ = 0.1 V, *V*_G_ = 0 V) as a function of optical irradiation power density at various wavelengths: blue, 255 nm; green, 370 nm; pink, 405 nm. (**b**) Source-drain voltage dependence of the photoresponsivity and detectivity for the NMe_2_-GQD@GFET hybrid photodetector (*V*_G_ = 0 V, 405 nm, 35 nW cm^−2^).

Figure 3a presents the optical power density-dependent external photoresponsivity *R*_ext_, and the specific detectivity D* of the NMe_2_-GQD@GFET hybrid photodetector at a fixed bias of 0.1 V under light irradiation at wavelengths of 255, 370, and 405 nm. D* (D* = (*∆f A*_PD_)^1/2^ *R*_ext_/*i*_n_, where *A*_PD_, *∆f* and *i*_n_ respectively stand for the area of the photodetector channel, electrical bandwidth, and noise current)^23, 24^ represents the capability of detecting low-level light signals. The noise current of photodetectors is dominated by the shot noise, but the noise current in the shot-noise limit is given as *i*_n_ = (2*eI*_dark_ *∆f*)^*1/2*^, where *e* and *I*_dark_ respectively denote the electron charge and the dark current^24^. Therefore, D* in the shot-noise limit is calculable by the expression: *R*_ex_ *A*_PD_^1/2^/(2*eI*_dark_)^*1/2*^ ^23–25^, which has been used to estimate the D* of photodetectors in earlier studies^23, 24, 26, 27^. The NMe_2_-GQD@GFET hybrid photodetector exhibited high photoresponsivity and detectivity in the deep UV region. *R*_ext_ and D* respectively reached ca. 1.5 × 10^4^ A W^−1^ and ca. 5.5 × 10^11^ Jones at 255 nm. The maximum values of *R*_ext_ and D* are comparable to those reported for other high performance UV photodetectors (Table S1)^6, 28–37^. The increased *R*_ext_ and D* toward the shorter wavelength is consistent with the fact that the absorption of the NMe_2_-GQDs is enhanced at high-energy wavelengths. The photocurrent increases linearly with the source–drain bias. High *R*_ext_ and D* is achieved when the bias voltage is increased and the incident optical power density is decreased (Fig. 3b). The decrease in *R*_ext_ and D* with the incident optical power density is explainable by consideration of the following reason. As more photogenerated electrons are injected into the graphene channel, the corresponding holes left in NMe_2_-GQD layers weaken the original internal field near the NMe_2_-GQD/graphene interface built by the Fermi-level alignment. The ability in charge separation declines with reduced interfacial electric field, leading to decreased photocurrent as the incident optical power density increases. Therefore, a reduced injection of electrons causes *R*_ext_ and D* to decrease.

**Figure 4:**
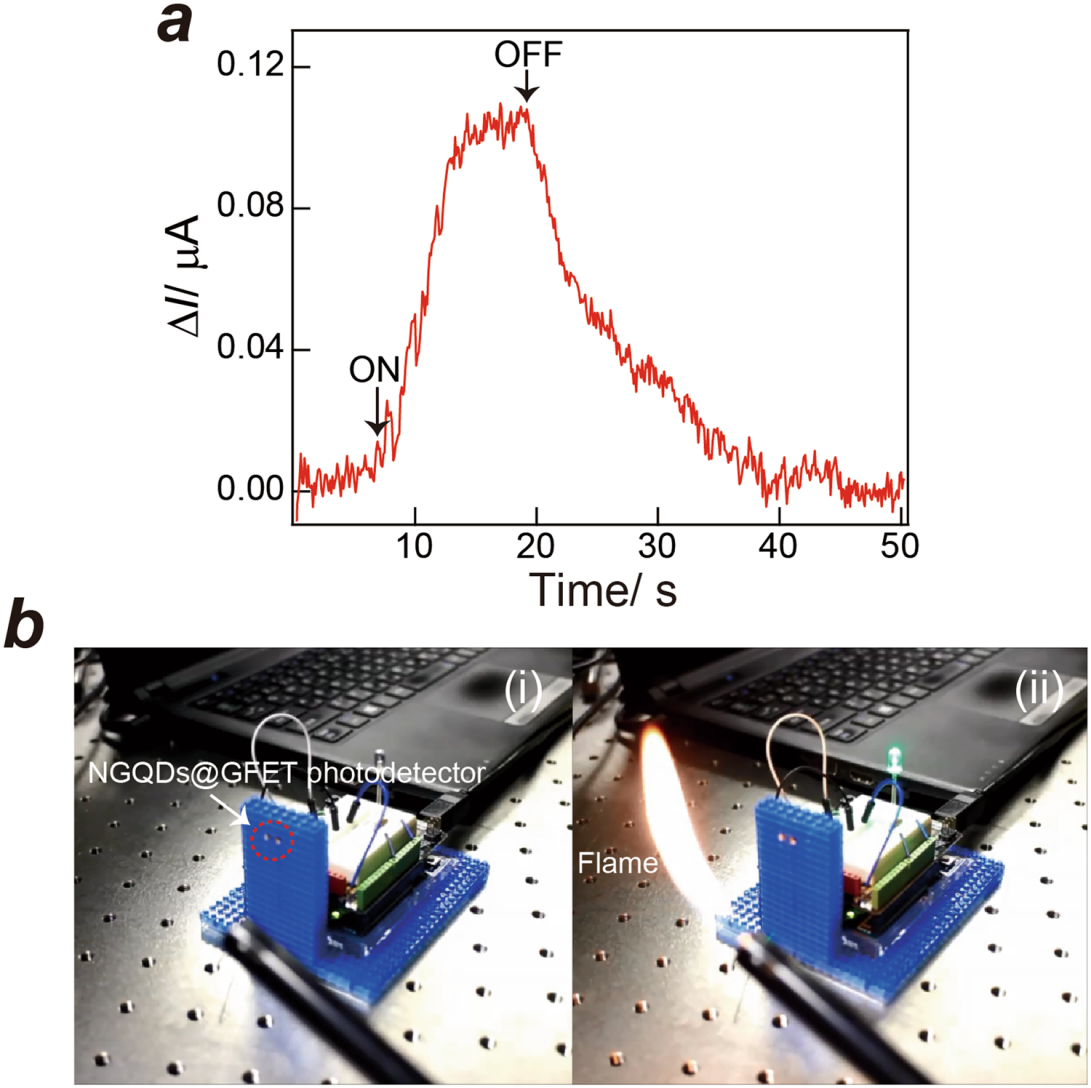
(**a**) Change in the photocurrent under UV light irradiated from a flame (the distance between the photodetector and flame was ca. 1 m. (**b**) Digital images (snapshots from Movie S1, Supplementary Information) showing the operation of the flame alarm using a NMe_2_-GQD@GFET hybrid photodetector. (i) Schematic of the prototype flame alarm. (ii) The green-LED of the fire alarm was turned on when the flame was detected.

Finally, the practical application of the NMe_2_-GQD@GFET hybrid photodetector to flame detection is demonstrated. Figure 4a presents the temporal change in the photocurrent upon illumination by light from a gas match from a distance of ca. 1 m. A clear change of the photocurrent was observed with switching of the gas match on and off, which confirms that the photodetector is useful for flame detection. Figure 4b depicts digital images of the prototype flame alarm mounted on a front panel consisting of “nanoblocks” in conjunction with the open-source Arduino Uno electronics platform.

A movie showing operation of the flame alarm is given in the Supplementary Information (Movie S1). When the photocurrent from the photodetector is increased to the threshold value of the photocurrent set in the program that controls the detectable distance, the green LED is turned on. The flame alarm LED was turned respectively off (Fig. 4b(i)) and on (Fig. 4b(ii)) in the absence and in the presence of a flame. Our flame alarm based on the NMe_2_-GQD@GFET hybrid photodetector is well operated. These results indicate that the proposed NMe_2_-GQD@GFET hybrid photodetector offers great potential for use in practical devices.

In summary, flexible UV photodetectors based on graphene/nitrogen-functionalized graphene quantum dot hybrid were produced using dimethylamine-functionalized GQDs with a large bandgap of ca. 3.3 eV. The hybrid photodetector exhibited high photoresponsivity and detectivity in the deep ultraviolet region. Its photoresponsivity and detectivity reached values exceeding 10^4^ A W^−1^ and 10^11^ Jones, respectively, at 255 nm. Moreover, the potential application for flame alarms was demonstrated. This hybrid photodetector is promising for use in future graphene-based photonic devices.

## Materials and Methods

### Materials

Graphite nanoparticles (GNPs; <50 nm; SkySpring Nanomaterials, Inc.) were used as a precursor for preparation of the NGQDs. N,N-dimethylformamide (DMF) as a solvent, and sulfuric acid and nitric acid as oxidizing agents were obtained from Wako Pure Chemical Industries Ltd.

### Synthesis of NMe_2_-GQDs

NMe_2_-GQDs were selected for the NGQD layer because their large energy gap (3.3 eV) enables visible-blind UV light detection. The NMe_2_-GQDs were prepared as follows. Oxidized GNPs (oGNPs) were obtained by refluxing GNPs in a 2:1 solution of concentrated H_2_SO_4_ and HNO_3_. 5 mg of the oGNPs were dispersed in 5 mL of DMF. The mixture was refluxed at 154.5 °C for 5 h. After cooling to room temperature, the suspension was dried using an evaporation system (Soltramini; Techno Sigma Corp.). The dried powder (NMe_2_-GQDs) was re-dispersed in water and was dialyzed for 3 days. After drying, the NMe_2_-GQD powder was re-dispersed in DMF.

### Photodetector device fabrication

Monolayer graphene sheets formed by chemical vapour deposition (CVD) onto a PEN substrate (188 μm) were obtained from Graphene Platform Corp. Gold source and drain electrodes (100 nm) were fabricated directly on the top of the graphene/PEN substrate using sputter deposition through a metal shadow mask. These devices had effective channel length *L* of 80 μm and a channel width *W* of 200 μm. NMe_2_-GQD dispersions were subsequently dropped directly onto the top of the graphene layer.

Measurements of the gate-voltage dependence on the photoresponsivity were taken using photodetectors fabricated by replacement of the PEN substrate with a Si substrate. The gate contact was deposited onto the back side of the Si wafer substrate. Monolayer graphene sheets formed by CVD onto a p-doped Si wafer (525 μm, 0.002 cm, 90 nm SiO_2_ layer) were obtained from Graphene Platform Corp. The source and drain electrodes were patterned directly on the top of the graphene/Si wafer using a conventional photolithography process with a poly(methyl methacrylate) resist. The contacts were formed with titanium and gold (15 nm/100 nm) using a sputtering deposition method, followed by a lift-off process. These devices were fabricated with *L* = 10 μm and *W* = 10 μm. After the gate contact was deposited onto the back side of the Si wafer, the device was diced into 1 × 1 cm substrates. The NMe_2_-GQD dispersions were dropped directly onto the top of the graphene layer.

### Photoresponse measurements

A light source was guided into the photodetector through a circular aperture with area *A*. Light-emitting diodes (LEDs) with different wavelengths were used as light sources to provide light with wavelengths of 255, 370, and 405 nm. A fibre probe, coupled to a monochromator with a deuterium lamp, was used for spectral response measurements. The optical power density through the aperture (*P*_opt_) was measured using a silicon photodetector (S130VC; Thorlabs, Inc.). The incident (*P*_inc_) power density was then calculated using the relation: *P*_inc_ = (*A*_det_ × *P*_opt_)/*A*, where *A*_det_ is the active area of the photodetector. Electrical measurements were taken using a system source meter (2602 A; Keithley Instruments Inc.) in conjunction with LabVIEW software. All measurements were conducted at room temperature in the ambient atmosphere.

### Instrumentation

X-ray photoelectron spectroscopy (XPS; Quantera SXM; Ulvac-Phi Inc.) measurements were performed using a monochromated Al K*α* radiation source (100 μm spot diameter) of 1486.6 eV under high-vacuum conditions. Scans were acquired in the fixed analyzer transmission mode with pass energy of 26 eV and a surface/detector takeoff angle of 45°. High-resolution transmission electron microscopy (HRTEM; EX-2000; Hitachi Ltd.) was used with accelerating voltage of 200 kV. Optical transmittance and reflectance in the visible/NIR region of the samples was recorded using a spectrophotometer (UV-3600; Shimadzu Corp.). Reflectance was measured at an incident angle of 5° using an additional attachment. Measurements of work function and shallow energy levels were taken using a photoelectron spectrometer (AC-2; Riken Keiki Co. Ltd.). Atomic force microscopy (AFM; NanoscopeV D3100; Veeco Instruments) was used to investigate the height profile.

### Data Availability

The datasets generated during and/or analysed during the current study are available from the corresponding author on reasonable request and with permission of Toyota Central R&D Laboratories, Inc.

## Electronic supplementary material

Supplementary Information

Supplementary Movie
