# Supplementary material for: 2D/0D graphene hybrids for visible-blind flexible UV photodetectors
Source: Sci Rep. 2017 Jul 17;7:5544. doi: 10.1038/s41598-017-05981-y (PMC5514071; doi:10.1038/s41598-017-05981-y)
Supplement: Supplementary file 1 — Supplementary Information [file 41598_2017_5981_MOESM1_ESM.doc]

**Supporting Information:**

2D/0D graphene hybrids for visible-blind flexible UV photodetectors

Hiroyuki Tetsuka*

Frontier Research-Domain, Toyota Central R&D Labs., Inc., 41-1 Yokomichi, Nagakute, Aichi 480-1192, Japan
E-mail: h-tetsuka@mosk.tytlabs.co.jp

**
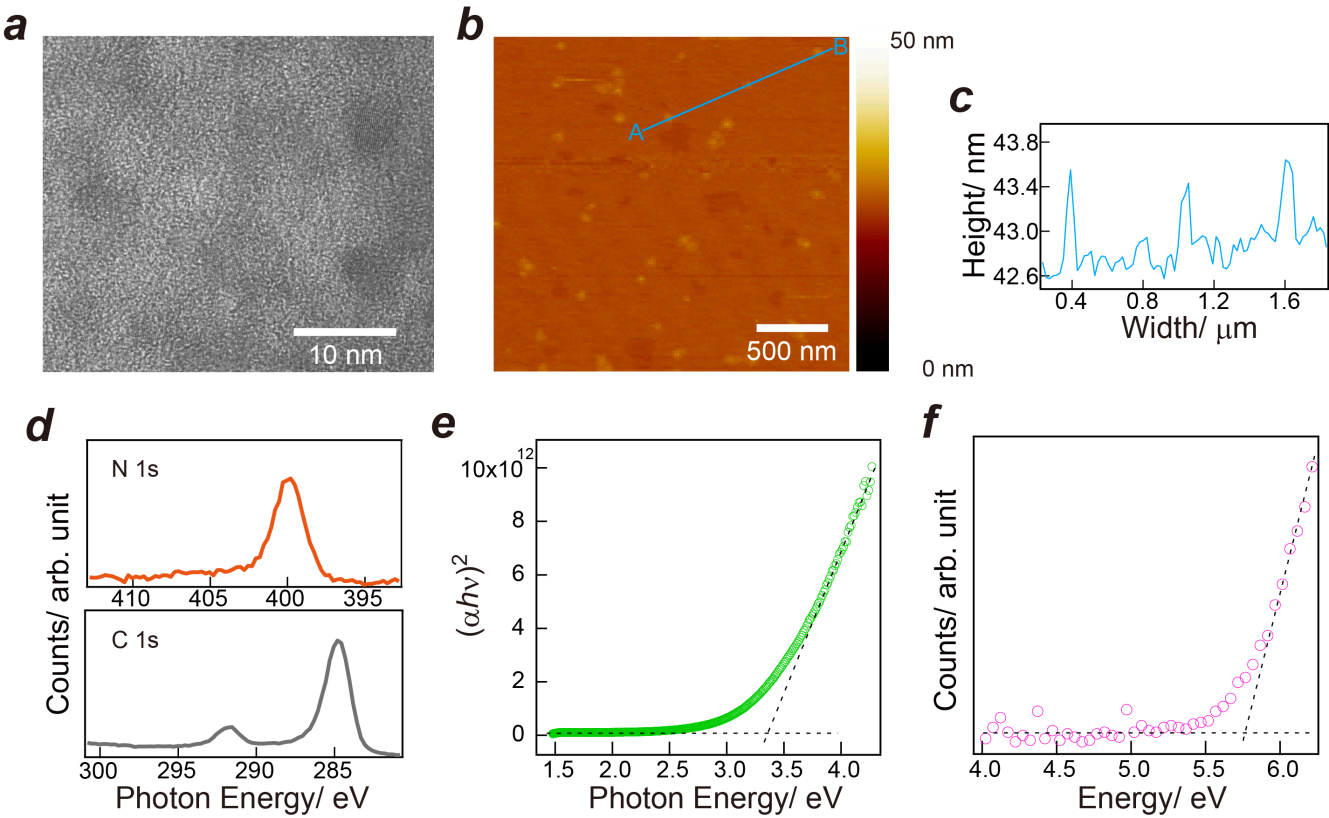
**

Fig. S1. Characterization of NMe_2_-GQDs: (a) HRTEM image of oxidized GNPs. (b) AFM image of NMe_2_-GQDs obtained by spin-casting the NMe_2_-GQD suspension on mica. (c) Height profile along the line A–B in (b). (d) C 1s and N 1s XPS spectra from NMe_2_-GQDs. (e) Tauc plot of NMe_2_-GQDs. (f) Photoelectron yield spectrum of NMe_2_-GQDs.

**Table S1.** Detectivity of 2D/0D graphene hybrid UV photodetectors and other various photodetectors.

| Material | Detectivity/ Jones (wavelength) |
| --- | --- |
| **This work** | **5×10^13^** **(255 nm)** |
| Graphene/ZnO QDs^S1^ | 5×10^13^ (335 nm) |
| ZnO nanowires^S2^ | 8×10^17^ (300 nm) |
| GaN PD^S3^ | 6.1×10^9^ (325 nm) |
| Al_0.2_Ga_0.78_N PD^S3^ | 1.2×10^9^ (325 nm) |
| GaN PD^S4^ | 1.7×10^13^ (<360 nm) |
| ZnMgO PD^S5^ | 3.1×10^11^ (330 nm) |
| SrTiO_3_ PD^S6^ | 2.2×10^11^ (355 nm) |
| MgO/ZnMgO MOS PD^S7^ | 1.2×10^13^ (366 nm) |
| ZnO-NiO PD^S8^ | 2×10^13^ (350 nm) |
| ZnO-polymer PD^S9^ | 6.5×10^12^ (390 nm) |
| VO_2_ microwire PD^S10^ | 1.5×10^14^ (360–400 nm) |
| TiO_2_-polymer PD^S11^ | 2×10^12^ (350 nm) |
| ZnO:PVK PD^S12^ | 3.4×10^15^ (360 nm) |

**Movie S1.** Operation of a flame alarm using a 2D/0D graphene hybrid UV photodetector.

References

S1. Shao, D., Gao, J., Chow, P., Sun, H., Xin, G., Sharma, P., Lian, J., Koratkar, N. A. & Sawyer, S. Organic–inorganic heterointerfaces for ultrasensitive detection of ultraviolet light *Nano Lett.* **15**, 3787−3792, doi: 10.1021/acs.nanolett.5b00380 (2015).

S2. Liu, X., Gu, L., Zhang, Q., Wu, J., Long, Y. & Fan, Z. All-printable band-edge modulated ZnO nanowire photodetectors with ultra-high detectivity. *Nat. Commun.* 5, 4007, doi:10.1038/ncomms5007 (2014).

S3. Monroy, E., Calle, F., Muñoz, E., Omnes, F., Gibart, P. & Muñoz, J. A. Al_x_Ga_1−x_N:Si Schottky barrier photodiodes with fast response and high detectivity. *Appl. Phys. Lett.* **73**, 2146, doi: 10.1063/1.122405 (1998).

S4. Chen, M. C., Sheu, J. K., Lee, M. L., Tun, C. J. & Chi, G. C. Improved performance of planar GaN-based *p*-*i*-*n* photodetectors with Mg-implanted isolation ring. *Appl. Phys. Lett.*, **89**, 183509, doi: 10.1063/1.2372767 (2006).

S5. Liu, K. W., Zhang, J. Y., Ma, J. G., Jiang, D. Y., Lu, Y. M., Yao, B., Li, B. H., Zhao, D. X., Zhang, Z. Z. & Shen, D. Z. Zn_0.8_Mg_0.2_O-based metal–semiconductor–metal photodiodes on quartz for visible-blind ultraviolet detection. *J. Phys. D: Appl. Phys.* **40**, 2765–2768, doi: 10.1088/0022-3727/40/9/014 (2007).

S6. Zhou, W., Jin, K., Guo, H., Ge, C., He, M. & Lu, H. Electrode effect on high-detectivity ultraviolet photodetectors based on perovskite oxides. *J. Appl. Phys.* **114**, 224503, doi: 10.1063/1.4845775 (2013).

S7. Zhu, H., Shan, C. X., Wang, L. K., Zheng, J. J., Zhang, Y., Yao, B. & Shen, D. Z. Metal−oxide−semiconductor-structured MgZnO ultraviolet photodetector with high internal gain. *J. Phys.Chem. C* **114**, 7169–7172, doi: 10.1021/jp101083n (2010).

S8. Kim, D. Y., Ryu, J., Manders, J., Lee, J. & So, F. Air-stable, solution-processed oxide *p*–*n* heterojunction ultraviolet photodetector. *ACS Appl. Mater. Interfaces* **6**, 1370–1374, doi: 10.1021/am4050019 (2014).

S9. Fang, Y., Guo, F., Xiao, Z. & Huang, J. Large gain, low noise nanocomposite ultraviolet photodetectors with a linear dynamic range of 120 dB. *Adv. Opt. Mater.* **2**, 348–353, doi: 10.1002/adom.201300530 (2014).

S10. Wu, J. M. & Chang, W. E. Ultrahigh responsivity and external quantum efficiency of an ultraviolet-light photodetector based on a Single VO_2_ microwire. *ACS Appl. Mater. Interfaces* **6**, 14286–14292, doi: 10.1021/am503598g (2014).

S11. Zhu, H. L., Choy, W. C. H., Sha, E. I. & Ren, X. Photovoltaic Mode ultraviolet organic photodetectors with high on/off ratio and fast response. *Adv. Opt. Mater.* **2**, 1082–1089, doi: 10.1002/adom.201400227 (2014).

S12. Guo, F., Yang, B., Yuan, Y., Xiao, Z., Dong, Q., Bi, Y. & Huang, J. A. A nanocomposite ultraviolet photodetector based on interfacial trap-controlled charge injection. *Nat. Nanotechnol.* **7**, 798–802, doi: 10.1038/nnano.2012.187 (2012).
